# An Analysis of Transient Overvoltages during the Energization of Electric Ship Propulsion Systems

**DOI:** 10.1155/2015/458068

**Published:** 2015-07-09

**Authors:** Morris Brenna, Federica Foiadelli, Dario Zaninelli

**Affiliations:** Department of Energy, Politecnico di Milano, Via La Masa 34, 20156 Milano, Italy

## Abstract

This paper addresses the resonance phenomena that can occur in an isolated distribution system during transient events such as repeated energizations or power converter switching. In particular, the aim of this study is to analyze the energization of an onboard radial distribution system installed on an electric ship and to determine how the various leakage parameters that can cause resonance problems such as high peak overvoltages when the circuit breaker is closed are relevant. The paper presents a detailed model of whole distribution system, which is validated using infield measurements that refer to a real case in which these events damaged the ships transformers, causing it to be removed from duty.

## 1. Introduction 

A ship can be considered a standalone system able to perform all of the necessary duties without the assistance of external entities [1]. All of these duties, which include propulsion, lighting, and conditioning, require electricity. For this reason, the power plant on the ship must be able to ensure that the right amounts of energy and power are available for the ship to perform these functions competently [2].

In recent years, thanks to progress in electrical engineering, electric systems have become more common in ships. They will replace all of the preceding systems for all functions, more or less, from energy generation to its transmission and utilization. A lack or inefficiency, even a partial one, of or in the electrical system of a ship is unacceptable because it limits the ship's operating efficiency. Moreover, electricity is necessary in emergency situations. It is clear that the main characteristics of a ship's electrical system are reliability, which is the assurance that each piece of equipment will function even in the worst conditions, and duty continuity, which is assurance, especially in emergency conditions, that the electrical supply will be sufficient at least for the apparatus required for the ship's security.

**Figure 1:**
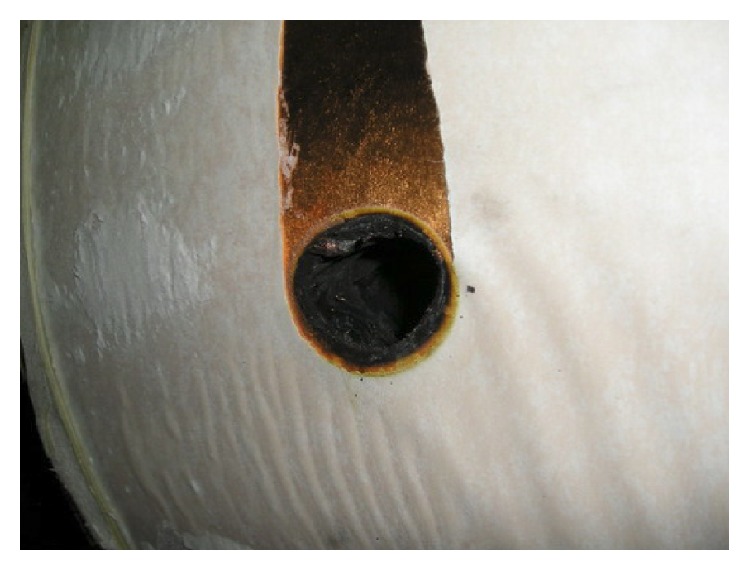
The effect of repetitive overvoltages on a transformer winding.

While reliability is focused on individual components, duty continuity is a property of the electrical system as a whole. Because of this requirement, attention is paid to problems that can affect the system's functionality, such as resonance phenomena, which can be very dangerous in an electrical system because they can cause high overvoltages that can damage installed appliances [3, 4].  Figure 1 shows an example of the effect of repeated overvoltages on a transformer winding.

In an example reported in [5], the catastrophic failure of a capacitor in the aft harmonic filter room on the RMS Queen Mary II is described as consequence of excessive harmonic distortion in the 11 kV distribution network.

Transient events are due to oscillations among the conservative components connected, or in any case present, in an electric circuit, and they generally occur for two reasons. The first one is the presence of voltage or current harmonics in the circuit at a frequency close to the resonance frequency. The second reason is related to the transient events that cause the system to oscillate at its natural frequency [6, 7].

It is not always possible to know the resonance frequency because the exact values of the RLC parameters and how they are connected in the circuit are unknown. Many parameters, including the series and magnetization inductance of the transformers and the capacitance of the power factor corrector, are well known because they are foreseen during the design process [8]. However, a large number of parameters are unpredictable because they depend on actual installations of various appliances and connecting cables and their working conditions [9]. In fact, these factors can cause leakage effects, such as series inductance and line to ground capacitance, which can increase the natural oscillation frequency of the system.

The aim of this paper is to analyze the energization of a radial distribution system installed on board an electric ship to identify the relevance of the various leakage parameters that can affect the resonance problem.

The classic electromagnetic models available in the scientific literature describe the stability of the electrical system; therefore, they are suitable for the low frequency analysis [10, 11].

This work presents a detailed model of the high frequency transients in the system and validates it using experimental measurements. The critical aspects are analyzed using simulations performed under many different network conditions.

## 2. The All-Electric Ship

Ship power systems require high levels of reliability because their operation is closely linked to human safety [12]. The increased use of electric propulsion leads to an increased installed generating capacity, to the use of different voltage levels, and, in general, to more complex systems.

Because the demand for electricity is typically comparable to the generation capacity and ship systems are isolated, it becomes obvious that, compared to terrestrial interconnected power systems, these systems present new challenges [13].

The future of electric ships depends on radical innovations and architectural transformations in the electrical power systems of naval ships. In an electric ship, the value of integration can be enhanced by the standardization of interfaces in the building blocks and well-defined control and protection requirements. In traditional vehicles, turbines or diesel engines directly drive or supply power to propulsion systems, and energy for other vehicle systems comes from power takeoffs or other generators. The idea of an integrated power system (IPS) is to enable all of the energy generated to be used by all of the ship's systems. In fact, early IPS concepts showed that there is enough energy in the propulsion system to supply a traditional ship's service needs, allowing dedicated ship service generators to be eliminated for significant fuel savings [14].

The transition from mechanical propulsion to electrical propulsion has caused dramatic changes in ship power systems. In traditional ships equipped with mechanical propulsion systems, electric energy is generated at a low voltage, typically 400 V, 50 Hz or 440 V, 60 Hz. In all-electric ships (AESs), electricity is generated at a higher voltage between 3.3 and 11 kV (at 50 Hz or 60 Hz) [15]. Although, according to terrestrial power standards, these voltages are in “medium voltage” range, in ships, they are commonly referred to as “high voltage” [16].

## 3. A Model of the Distribution System

**Figure 2:**
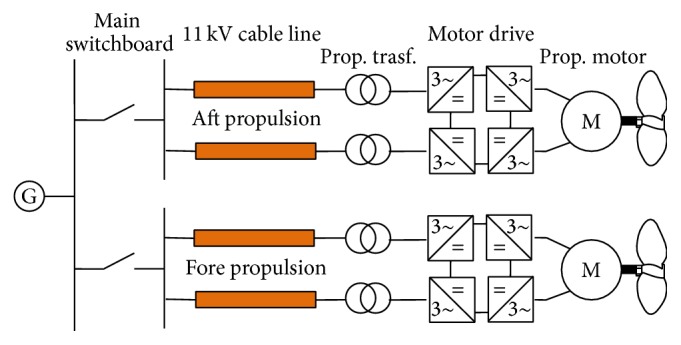
A propulsion system.

This section presents a model of the distribution system of an electric ship that considers the entire energy chain, from the main switchboard to the propulsion converter, as shown in Figure 2.

Particular attention has been paid to models of the circuit breakers installed in the main switchboard, MV line cables, and power transformer. The other devices, including the equivalent network upstream of the switchboard, have negligible effects on the fast transient phenomena that are the objects of this study because synchronous generators are characterized by higher transient and subtransient synchronous reactance than cables. Therefore, they can affect low frequency transients but do not impact high frequency quantities.

The model was implemented using ATP/EMTP, which is a program for simulating the electromagnetic transients of electric and electronic circuits.

### 3.1. The Main Switchboard

**Figure 3:**
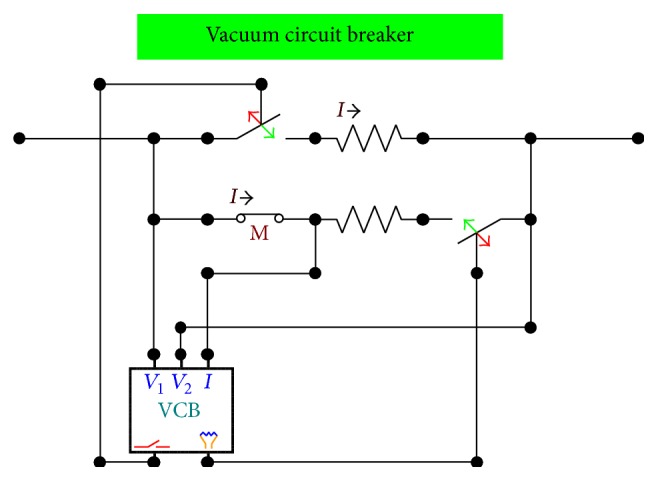
A model of the vacuum circuit breaker.

The study supposes that the main switchboard is supplied by an equivalent MV network modeled using an equivalent Thevenin circuit. After the MV Busbar, each line is protected by a typical vacuum circuit breaker of the type employed in MV distribution systems. A functional model of the vacuum circuit breaker has been derived from the international literature [17–20]. The final model (Figure 3) implements the following features:

- arc resistance when it is switched off;
- reignition of the arc when it is switched off;
- chopping of the current when it is switched off;
- asynchronous closing and opening of the three poles.

### 3.2. The Distribution System

**Figure 4:**
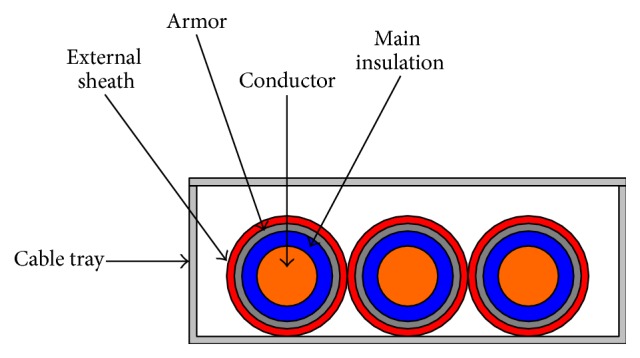
MV power line installed onboard.

A lumped parameter model is employed for the MV distribution cables connecting the power transformer to the main switchboard. To better describe the behavior at the highest frequencies, the cable line has been subdivided into many sections, each with a maximum length of 10 m. The model takes into account a three-phase line consisting of three single core armored cables laid in a metallic cable tray, as shown in Figure 4.

In each line section, all of the self and mutual inductances and capacitance of the cores and armor of the cables are considered.

**Figure 5:**
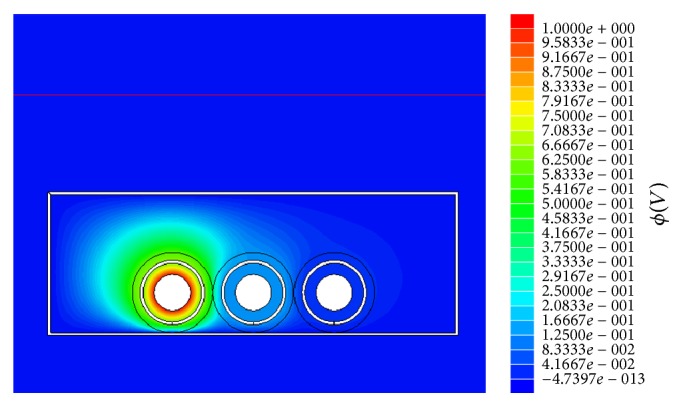
One screenshot of the FEM analysis for determining the parameters.

To determine the correct parameters for the line model based on the self and mutual inductances, resistances, and capacitance, a Finite Element Method (FEM) analysis was performed (Figure 5).

The results of the FEM analysis are shown in Tables 1 and 2. The self and mutual values of the series and shunt parameters are reported.

**Figure 6:**
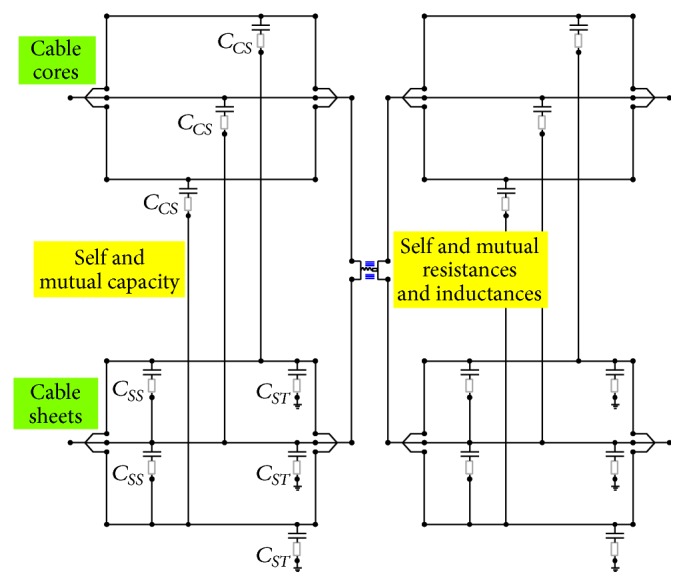
The final model for a section of the MV cable.

Considering the above parameters and observing that much of the mutual capacitance are negligible, the final model of a section of the MV cable is the one shown in Figure 6.

From Table 2, it is then possible to calculate the capacitance of the capacitors in the model shown in Figure 6:

- *C*_*CS*_ = 0.00143 *μ*F,
- *C*_*SS*_ = 0.00023 *μ*F,
- *C*_*ST*_ = 0.0006452 *μ*F.

### 3.3. The Propulsion Transformer

The propulsion transformer considered in this system is of the type typically used in multilevel power converters [21].

The secondary side of the propulsion transformer is split into main and shift windings with a middle tap to provide the correct voltage phase shift for the propulsion power converters to reduce the voltage and current harmonics. Therefore, the model of its circuit was derived using the classical theory of three-phase, three-winding transformers.

The EMTP model during transients is described in the following sentences. Incoming disturbances from the generators of the distribution network plus the transformer itself are usually represented by a voltage source behind *R*-*L* branches. Because the transformer inductances tend to filter out high frequencies, using such a low frequency *R*-*L* circuit appears to be reasonable. Therefore, the transformer is modeled in EMTP by a star circuit, which uses the matrices [*R*] and [*L*]^−1^ in the alternative equation:

(1) $$\begin{array}{c} {\left[\;L\;\right]}^{-1}\left[\;v\;\right]={\left[\;L\;\right]}^{-1}\left[\;R\;\right]\left[\;i\;\right]+\left[\;\frac{di}{dt}\;\right] \end{array}$$

for the transient solution. [*R*] and [*L*]^−1^ are determined and obtained using the BCTRAN routine.

**Figure 7:**
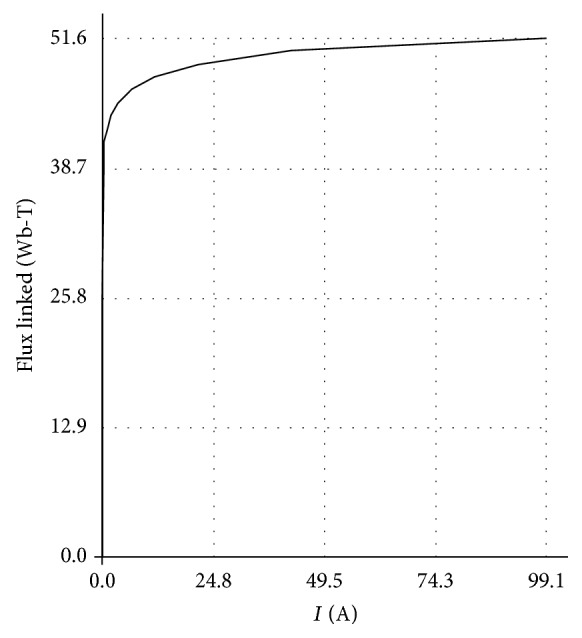
The saturation curve for the converter transformers considered in this study.

The exciting curve that is highly voltage-dependent above the “knee” of the saturation curve *λ* = *f*(*i*) is also considered.  Figure 7 shows that the magnetization curve considered is a typical BH curve for a modern high-voltage transformer with a grain-oriented steel core. The knee occurs at approximately 1.1 to 1.2 times the rated flux. The value of the incremental inductance *dλ*/*di* is fairly low in the saturated region and fairly high in the unsaturated region. The magnetization current in the unsaturated region can be included in [*L*]^−1^.

**Figure 8:**
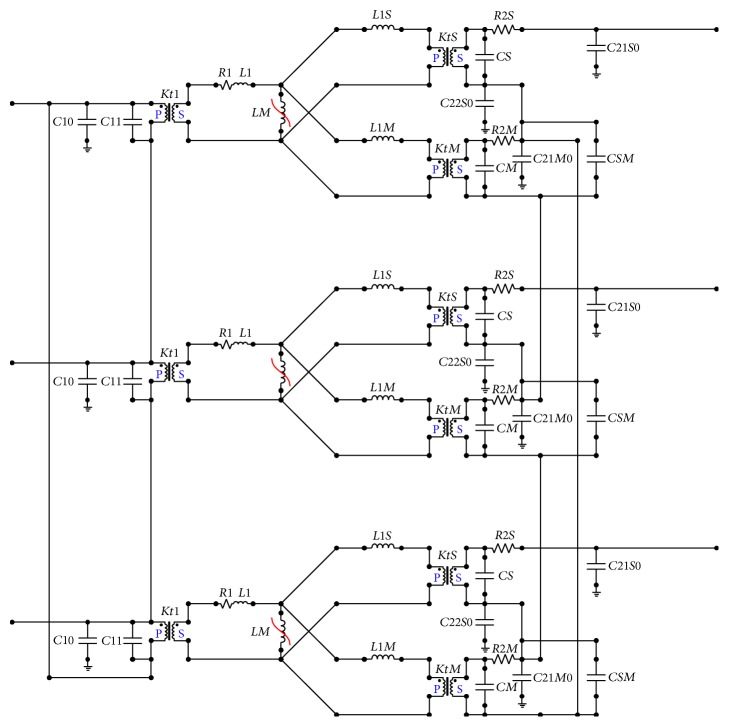
The equivalent circuit for the converter transformers considered in this study.

The final equivalent circuit is shown in Figure 8, in which all of the series and shunt parameters are represented.

Extra nonlinear branches are needed to model saturation effects, and extra resistance branches are needed to model core losses. The extra capacitance added in the transformer model has been evaluated using a dedicated FEM analysis. They represent the leakage capacities between the various windings and between the windings and the ground.

For each phase, the shift coil is represented in the upper part, while the main secondary coil is placed in the lower part.

The primary side elements are ones upstream of the magnetizing nonlinear inductance *LM*.

The values of the primary parameters of the transformer's equivalent circuit are reported in Table 3.

### 3.4. The Propulsion Converters

**Figure 9:**
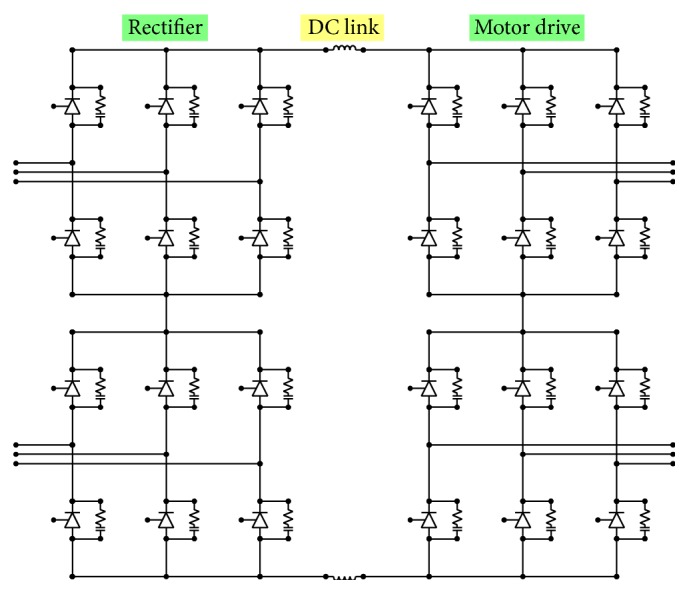
The model of the propulsion motor drive.

The model of the propulsion motor drive is shown in Figure 9.

The propulsion motors are supplied with electricity through a dedicated synchro converter that works as a motor drive by providing a variable voltage and frequency. Although this converter does not work during the energization and deenergization phases of the propulsion system, it must be considered in the study of transients due to the presence of reactive elements such as filter inductances and snubber capacitance that are always connected. In fact, these conservative elements influence the natural oscillation frequency of the system and, consequently, the resonance phenomena.

The propulsion motors are double-star synchronous machines; therefore, they must be supplied by a two-phase-shifted inverter.

The DC link is created by two-phase-shifted thyristor rectifiers connected to the secondary side of the propulsion transformers.

Because it is necessary to have a 24-pulse reaction in the main ship distribution system from the two motor drives, there must be a shifter winding on the secondary side of each propulsion transformer.

## 4. Model Validation through Measurements in the Field

The model is validated by comparing the results of the simulation with measurements of a real installation. The test system has these particular characteristics:

- cables lengths of 83 m and 79 m (long cables);
- asynchronous triggering of the circuit breaker at time pole 1: 13 ms, pole 2: 11.5 ms, and pole 3: 11 ms;
- cable armor that is grounded on both sides.

Validating the model by comparing the simulation results and measurements can be complex due to the difficulty of simulating the timetable of the various events and the EMI disturbances that affect the measurement instruments and probes (Rogowski coils). However, qualitative correspondences can be found between them.

**Figure 10:**
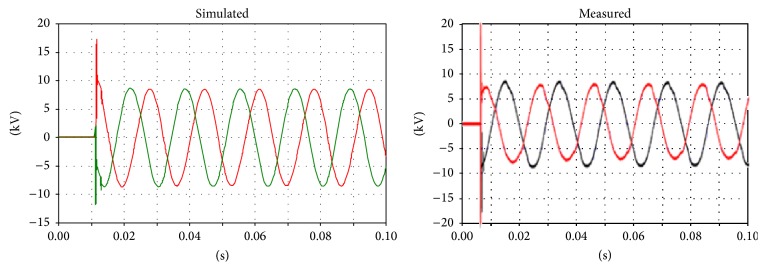
Voltage on the primary side of the transformer; phases U and V.

**Figure 11:**
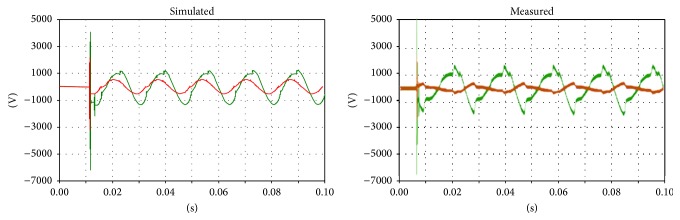
Voltage on the secondary side of the transformer; green: main coil, red: tap coil.

**Figure 12:**
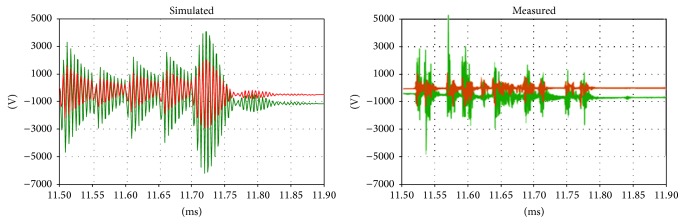
Details of the transient voltages on the secondary side of the transformer during the inrush; green: main coil, red: tap coil.

Regarding the voltages on the primary and secondary sides, comparison is difficult because the instant closure of the circuit breaker dramatically affects the transient phenomena. However, correspondences between the measurements and simulations can be found between the voltages on the primary and secondary sides (Figures 10, 11, and 12).

## 5. An Analysis of the Critical Configuration

To evaluate the critical configuration of the onboard distribution system, a test system is simulated in the EMTP environment. The test system consists of equivalent generator that feeds the main switchboard, a three-pole vacuum circuit breaker, a three-phase cable line, a power transformer, and a motor drive power converter. The analysis focuses on possible resonance phenomena that can occur during the energization of the distribution system under no-load conditions. In particular, these phenomena can occur when inductive loads such as power transformers or large asynchronous motors are supplied by long cable lines. The resonance condition depends on the values of the conservative elements that characterize the power system, including the cable capacitance and the transformer leakage and magnetizing inductances. The variation of the natural oscillation frequency due to the variation of the leakage parameters determines which resonance phenomena occur inside the transformer. These can include the origination of overvoltages during transients such as turning on the system during its energization phase, especially if the transformer is in a no-load state, that is, without any damping due to a load [22]. Therefore, the detailed model of the various components presented above has been used.

To highlight the relationship between the leakage parameters and overvoltages, the energization of the distribution system is studied in detail and simulated for two cable lengths that correspond to two different transformer positions. The voltages on the primary and secondary sides are recorded for each length.

For a more realistic simulation, the asynchronous actuation of the three contacts of the vacuum circuit breaker occurs at 13 ms, 11.5 ms, and 11 ms.

**Figure 13:**
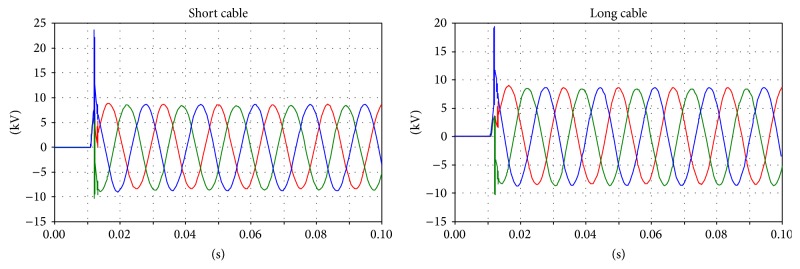
The primary side voltages during the inrush.

**Figure 14:**
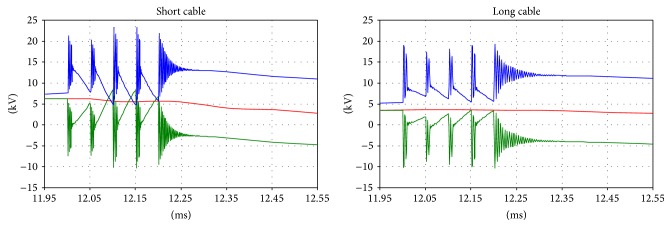
Primary side voltages: detail of the inrush.

**Figure 15:**
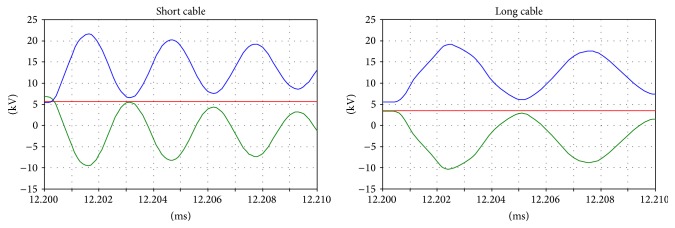
Primary side voltages: the transient oscillation frequency during the inrush.

**Figure 16:**
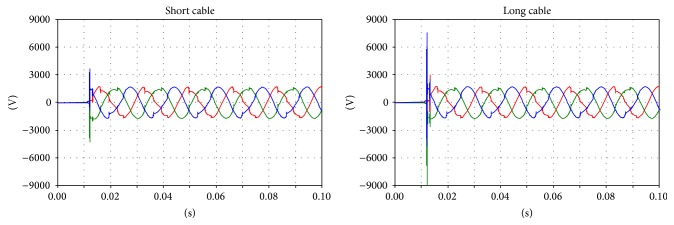
The secondary side voltages during the inrush.

The results of the simulation are shown in Figures 13, 14, 15, and 16.

It is notable that there are no differences in the overvoltages on the primary side, but this is not true for the secondary side. The change in the natural oscillation frequency can cause resonance phenomena between the primary and secondary sides. In this case, during the inrush, higher overvoltages can occur and can result in high stress on the system isolating the cables and transformers.

**Figure 17:**
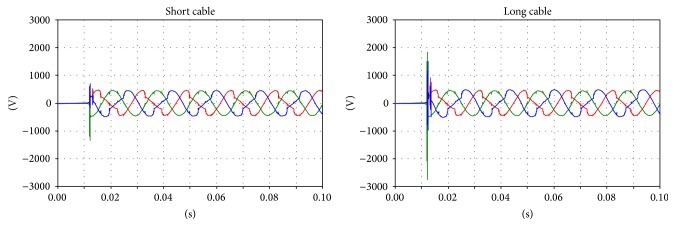
The tap coil voltages during the inrush.

**Figure 18:**
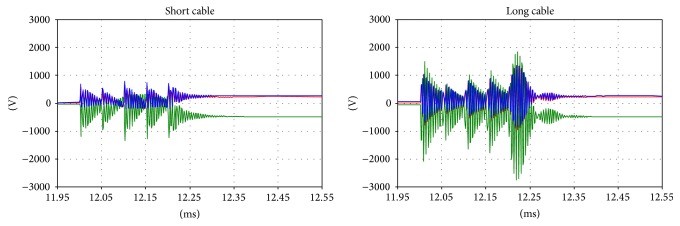
The transient voltages in the tap coil during the inrush.

**Figure 19:**
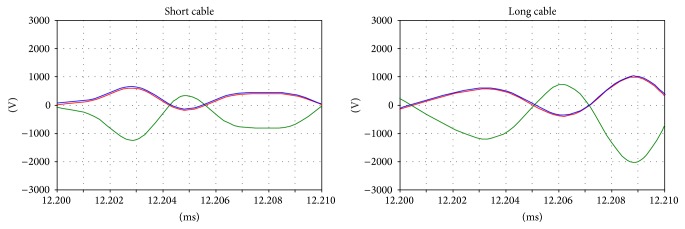
Details of the transient voltages in the tap coil during the inrush.

In particular, by analyzing the behavior of the shift coil (Figures 17 and 18), it is possible to observe that the variation in the natural oscillation frequency (Figure 19) produces transient phenomena that lead to high frequency overvoltages.

During a cruise, the power system's configuration continuously changes due to variations in the amount of power requested by the users and the propulsion system. Therefore, the number of times part of the system is switched on or off is high, which subjects the secondary coils to repetitive high-voltage peaks that were not foreseen during the design phase. In fact, they depend on many uncertain factors, including the cables' lengths and paths; therefore, they can be evaluated only after the system has been constructed.

This continuous stress on the insulation can degrade the properties that determine the electric arc inside the windings and result in the subsequent destruction of the transformer.

## 6. Conclusions

In isolated power systems, the need for load balancing requires many reconfigurations of the system and, consequently, the circuit breaker must be switched on and off many times and, under certain conditions, transient overvoltages can occur due to resonance phenomena. A critical consequence of these problems is the possibility of a fire hazard, which has serious safety issues, especially on board a ship.

Because damage to transformers installed on cruise ships has actually occurred, this paper aimed to determine the conditions that can lead to dangerous phenomena. Therefore, a model of a typical distribution system installed on a cruise ship was created and validated using infield measurements.

The many simulations performed determined the most critical conditions with particular reference to MV distribution cable lengths. In fact, the same system can be installed in a different part of the ship, that is, in the fore or aft section, resulting in different cable lengths.

The model has been useful to verify the fact that overvoltages that are too high can occur when the cable length varies from that of a well-known system. The results show that it is possible for resonance phenomena to lead to repetitive high overvoltages, damaging devices such as transformers and power converters.

Once these problems arise, different solutions can be used; for example, it is possible to split one cable into two parallel conductors, increase its cross section, and define a different path.

## Figures and Tables

**Table 1:** The series parameters for the MV cable line.

	Core 1		Core 2		Core 3	
	*R* [Ω/m]	*L* [H/m]	*R* [Ω/m]	*L* [H/m]	*R* [Ω/m]	*L* [H/m]

Core 1	**0.750 · **10^−3^	**1.96 · **10^−3^	0.673 · 10^−3^	1.48 · 10^−3^	0.663 · 10^−3^	1.21 · 10^−3^
Core 2	0.673 · 10^−3^	1.48 · 10^−3^	**0.771 · **10^−3^	**1.88 · **10^−3^	0.711 · 10^−3^	1.46 · 10^−3^
Core 3	0.663 · 10^−3^	1.21 · 10^−3^	0.711 · 10^−3^	1.46 · 10^−3^	**0.828 · **10^−3^	**1.93 · **10^−3^
Screen 1	0.682 · 10^−3^	1.81 · 10^−3^	0.673 · 10^−3^	1.48 · 10^−3^	0.663 · 10^−3^	1.21 · 10^−3^
Screen 2	0.673 · 10^−3^	1.48 · 10^−3^	0.703 · 10^−3^	1.72 · 10^−3^	0.711 · 10^−3^	1.46 · 10^−3^
Screen 3	0.663 · 10^−3^	1.21 · 10^−3^	0.711 · 10^−3^	1.46 · 10^−3^	0.760 · 10^−3^	1.77 · 10^−3^

	Screen 1		Screen 2		Screen 3	
	*R* [Ω/m]	*L* [H/m]	*R* [Ω/m]	*L* [H/m]	*R* [Ω/m]	*L* [H/m]

Core 1	0.682 · 10^−3^	1.81 · 10^−3^	0.673 · 10^−3^	1.48 · 10^−3^	0.663 · 10^−3^	1.21 · 10^−3^
Core 2	0.673 · 10^−3^	1.48 · 10^−3^	0.703 · 10^−3^	1.72 · 10^−3^	0.711 · 10^−3^	1.46 · 10^−3^
Core 3	0.663 · 10^−3^	1.21 · 10^−3^	0.711 · 10^−3^	1.46 · 10^−3^	0.760 · 10^−3^	1.77 · 10^−3^
Screen 1	**0.801 · **10^−3^	**1.81 · **10^−3^	0.673 · 10^−3^	1.48 · 10^−3^	0.663 · 10^−3^	1.21 · 10^−3^
Screen 2	0.673 · 10^−3^	1.48 · 10^−3^	**0.821 · **10^−3^	**1.72 · **10^−3^	0.711 · 10^−3^	1.46 · 10^−3^
Screen 3	0.663 · 10^−3^	1.21 · 10^−3^	0.711 · 10^−3^	1.46 · 10^−3^	**0.879 · **10^−3^	**1.77 · **10^−3^

**Table 2:** The self and mutual capacitance matrix of the MV cable line.

	Core 1	Core 2	Core 3	Screen 1	Screen 2	Screen 3
Core 1	0.000143	0	0	−0.000143	0	0
Core 2	0	0.000143	0	0	−0.000143	0
Core 3	0	0	0.000143	0	0	−0.000144
Screen 1	−0.000143	0	0	0.000231	−0.000023	0
Screen 2	0	−0.000143	0	−0.000023	0.000247	−0.000023
Screen 3	0	0	−0.000144	0	−0.000023	0.000231

**Table 3:** Values of the primary transformer parameters.

Parameter	Value	UM
*Kt1 *	1 : 1	Turn/turn
*KtS *	262 : 8	Turn/turn
*KtM *	262 : 40	Turn/turn
*L1 *	20.64	mH
*L1S *	5.026	mH
*L1 M *	−0.7504	mH
*C10 *	0.001312	*μ*F
*C11 *	31 · 10^−6^	*μ*F
*CS *	0.012	*μ*F
*C21S0 *	175 · 10^−6^	*μ*F
*C22S0 *	175 · 10^−6^	*μ*F
*CM *	415 · 10^−6^	*μ*F
*CSM *	350 · 10^−6^	*μ*F
*C21 M0 *	660 · 10^−6^	*μ*F
*R1 *	0.1231	Ω
*R2S *	0.3408	mΩ
*R2 M *	3.594	mΩ
